# Rendezvous procedure to establish ureteral continuity from a delayed ureteral leak following pelvic surgery. A case report

**DOI:** 10.1016/j.ijscr.2020.10.014

**Published:** 2020-10-10

**Authors:** George Chang, Arshad A. Khan, Saher Sabri, Paul H. Sugarbaker

**Affiliations:** a Department of Urology, MedStar Washington Hospital Center, Washington, DC, USA; b Department of Interventional Radiology, MedStar Washington Hospital Center, Washington, DC, USA; c Center for Gastrointestinal Malignancies, MedStar Washington Hospital Center, 3629 Fulton St. NW, 20007, Washington, DC, USA

**Keywords:** Appendiceal mucinous neoplasms, Pseudomyxoma peritonei, Ureteral stent, Percutaneous nephrostomy, Partial sacrectomy, Ureteral trauma, Ureteral necrosis

## Abstract

**Background:**

Iatrogenic damage to the ureter as a result of an abdominal or pelvic surgical procedure is unusual. However, it does occur and the surgeon must be prepared to deal knowledgeably with the injury. Leaks that are recognized within the operating theater are managed, for the most part, at the same surgical procedure oftentimes with urologic consultation. A delayed leak presents unique problems in that direct access to the site of the leak is not possible except by a reoperative procedure. Delayed leaks present a clinical situation involving the urologist, interventional radiologist, as well as the surgeon.

**Methods:**

A patient who developed delayed urine leakage following a partial sacrectomy to remove recurrent mucinous appendiceal malignancy was studied. The leakage was controlled using a nephroureteral stent. Placement of the nephroureteral stent was made possible by the rendezvous procedure.

**Results:**

The sequence of procedures to reestablish ureteral continuity following a delayed leak are important in the successful placement of a nephroureteral stent. After establishing the site of the injury a percutaneous nephrostomy must be placed. Then, through the nephrostomy, a guidewire is placed in the ureter to be recovered and advanced into the bladder using a ureteroscope and grasping forceps. A nephroureteral stent is placed over the guidewire to bridge the gap and stent the ureteral defect.

**Conclusions:**

The rendezvous procedure can be successful a large percentage of the time with a delayed ureteral leakage. Successful recovery of a guidewire in the ureter by ureteroscopy requires a combined interventional radiology and urologic procedure.

## Highlights

• Delayed leak from a ureter can occur following pelvic surgery.
• Stenting the ureteral defect through the bladder or kidney is often unsuccessful.
• A rendezvous procedure may be successful to reestablish ureteral continuity.
• A sequence of collaborative efforts by urologist and interventional radiologist is required.

## 1 Introduction

Damage to the ureter with primary colon and rectal surgery is unusual. It has been recorded in less than 1% of the colorectal surgeries performed in the United States. If a genitourinary injury occurs, it involves the transection of a ureter, proximal ureteral injury or distal ureteral injury in approximately 50% of patients [1]. In cytoreductive surgery for peritoneal metastases, reoperations are frequently required [2,3]. The reoperative procedures are of benefit to patients if all of the peritoneal metastases visible to the radiologist preoperatively and the surgeon intraoperatively are removed [4]. In these reoperative procedures all tumor must be removed including that which is adjacent or adherent to the ureter. This places the ureter at risk. In most instances in reoperative surgery, placement of ureteral stents is indicated in order to minimize the possibility for iatrogenic ureteral injury. When these injuries do occur, almost always the damage is recognized in the operating room and the damage can be repaired with the placement of a ureteral stent, closure of the small defect in the ureter, coverage of the ureter with soft tissue, and generous percutaneous drainage. Ureteral transection may require cross ureteroureterostomy [5] or a reimplantation of the ureter into the bladder. In a minority of iatrogenic ureteral injuries, the leakage of urine occurs after the abdomen has been closed. These delayed leakages require the expertise of the interventional radiologist and urologist because, in the absence of reoperative surgery, direct access to the ureter is not possible. In this manuscript we report on the rendezvous procedure to restore continuity of proximal and distal ureter following delayed urine leakage. This patient presentation has been reported in line with the SCARE criteria [6]. The study was registered as a case report on the www.researchregistry.com website with UIN 6018. Written consent was obtained from the patient to write this case report.

## 2 Patient presentation

Spring of 2012. A 48-year-old woman began to complain of lower abdominal and pelvic pain that continued throughout that year.

March 2013. On a visit to her physician’s office an abdominal mass was palpated. A CT scan was performed revealing a large pelvic mass and fluid surrounding the right lobe of the liver.

March 20, 2013. Patient underwent open abdominal surgery. She had an appendectomy, hysterectomy, and bilateral salpingo-oophorectomy. A left ovarian mass 20 cm in diameter and a 4 cm appendiceal mucinous tumor were resected. The pathology showed metastatic moderately differentiated mucinous adenocarcinoma with a primary site within the appendix. The right ovary was also involved and tumor biopsies from the greater omentum were positive for malignancy.

March/April 2013. The patient underwent 3 cycles of FOLFOX chemotherapy which was tolerated poorly.

September 19, 2013. The patient underwent cytoreductive surgery with perioperative intraperitoneal chemotherapy (HIPEC) with intraperitoneal doxorubicin and mitomycin C combined with systemic 5-fluorouracil and leucovorin [7,8]. This was a 15-h surgical procedure which involved total parietal peritonectomy, greater omentectomy and splenectomy, left ureteral lysis, excision of the apex of the vagina, and total abdominal colectomy with end-ileostomy. The right pleural space was entered during the right subdiaphragmatic peritonectomy and was repaired as part of this surgical procedure. The patient’s postoperative stay and recovery was prolonged but without adverse events. Pathology revealed mucinous adenocarcinoma of low cellularity (less than 20% epithelial component). The histopathologic diagnosis was mucinous neoplasm from the appendix with changes induced by the neoadjuvant systemic FOLFOX chemotherapy.

May 23, 2017. CT abnormalities developed with stable CEA and CA19-9 tumor markers. A third surgical procedure was performed on May 23, 2017. This was a 9½ hour surgery which included the rectal stump and two-thirds of the residual vagina.

**Fig. 1:**
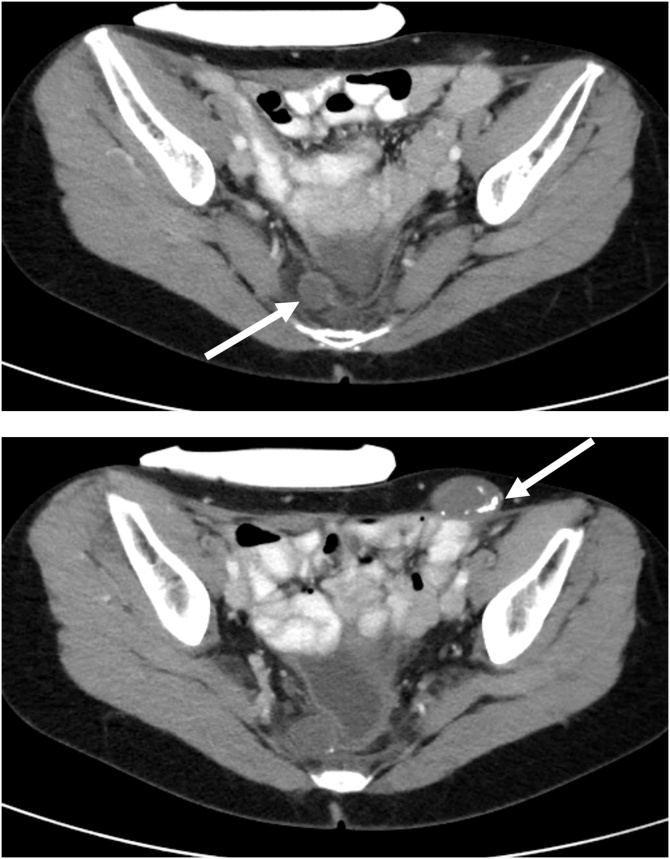
Top. CT cut through S4-S5. (05/18/2020) A tumor mass deep in the pelvis and adjacent to the bladder is depicted (indicated by an arrow). Bottom. CT cut through the upper coccyx. The pelvic mass demonstrated on CT appears to impinge and slightly deform the urinary bladder but is not invasive. The partially calcified mass in the left inguinal region is also depicted (indicated by an arrow).

February 12, 2020. A cystoscopy and cystogram were performed because of a mass seen on CT within the pelvis and on the right side of the bladder (Fig. 1, top and bottom). Also, bilateral inguinal masses were present and thought to be within the canal of Nuck [9].

**Fig. 2:**
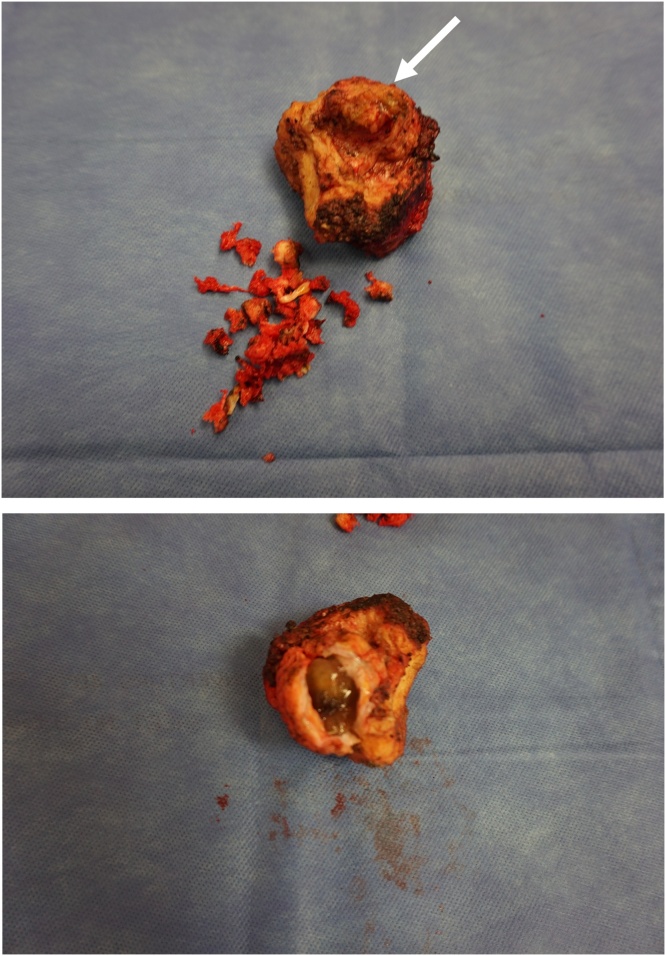
Top. Photograph of the anterior aspect of the resected specimen (06/23/2020). A tumor mass attached to the right side of the presacral fascia is shown (indicated by an arrow). The sacrum was resected from S4 down to and including the coccyx. No remnants of a ureter were present within the specimen. Bottom. The specimen is opened showing the mucin content of the presacral mass.

June 23, 2020. A fourth surgical procedure was planned to avoid reentry into the abdomen and pelvis to avoid an extensive adhesive process. The bilateral inguinal masses were resected without difficulty. A partial sacrectomy was performed through a perineal incision. The sacrum from the S4-S5 junction and coccyx were removed along with the underlying tumor mass (Fig. 2, top and bottom). The bladder was visualized during the dissection but was not thought to have been significantly traumatized. Blood loss was minimal. The ureters on the right and left were not encountered. No urine leak occurred during the surgical procedure. A closed-suction drain was left within the space previously occupied by the tumor mass and distal sacrum plus coccyx.

**Fig. 3:**
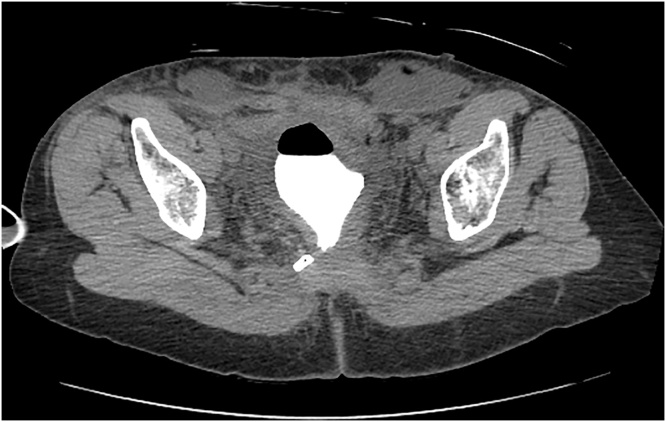
Cystogram (07/01/2020). A CT cystogram shows no contrast extravasation from the bladder. The closed suction drain remains in place. Lymphocoeles are present in the inguinal sites.

**Fig. 4:**
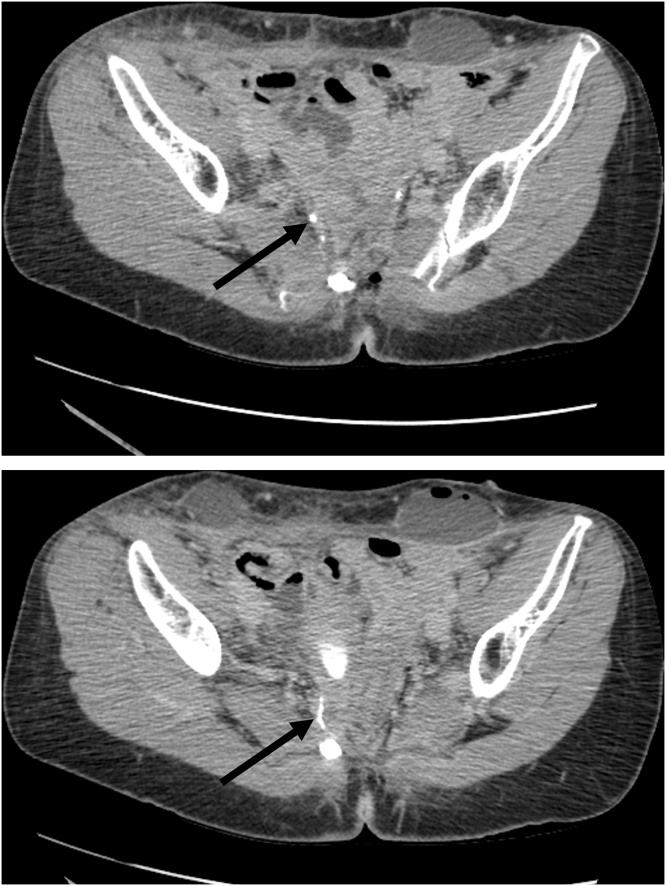
Top. CT with intravenous contrast (07/02/2020). CT slice through the pelvis at the level of S4. The distal right ureter is well demonstrated (indicated by an arrow). Bottom. CT with intravenous contrast. CT slice just beneath the right ureter depicted in Fig. 4, top shows a stream of contrast moving from the right ureter toward the closed suction drain in the presacral space (indicated by an arrow).

June 30, 2020. On the seventh day after surgery, abruptly a large volume of fluid was observed from the closed-suction drain that was placed in the presacral space at the time of surgery. Creatinine on this fluid was markedly elevated at 44 mg/μL. A cystogram was performed on July 1, 2020 which showed no leak from the bladder (Fig. 3). On July 2, 2020, using intravenous contrast (Omnipaque, GE Healthcare Inc., Marlborough, MA) a delayed CT of the kidneys and collecting system showed a leak from the distal right ureter into the presacral space (Fig. 4 top and bottom).

July 2, 2020. Cystoscopy was performed with an attempt to pass a stent up the right ureter. This was unsuccessful. Ureteroscopy of the right ureter suggested a 2 cm defect of the distal right ureter approximately 4 cm from the ureterovesical junction. The left ureter was normal.

July 2, 2020. The patient underwent percutaneous nephrostomy on the right site. A pyeloureterogram showed dye passing from the ureter into the presacral space.

**Fig. 5:**
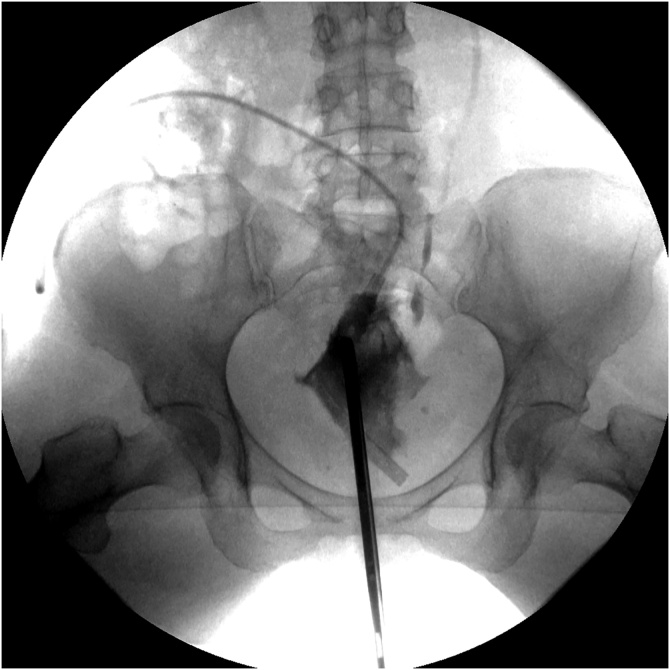
Rendezvous procedure (07/02/2020). A guidewire was passed through the right nephrostomy down the ureter to the ureteral leak and left in place. Cystoscopy and then ureteroscopy were performed. The guidewire in the right ureter was grasped through the ureteroscopy and pulled into the bladder.

**Fig. 6:**
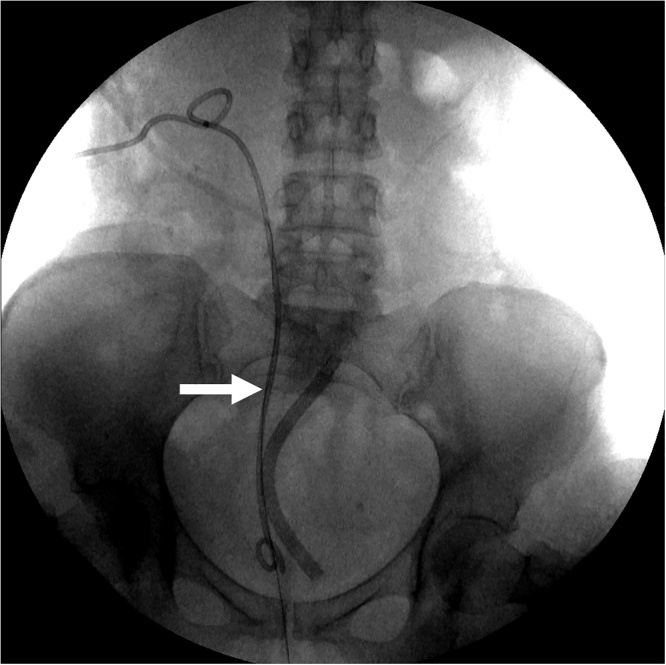
Placement of a ureteral stent from the right kidney into the bladder (07/02/2020). Using the guidewire, a nephroureteral stent was passed into the right kidney, down the right ureter and past the ureteral leak into the bladder (indicated by an arrow). The closed-suction drain in the operative site remained in place.

July 7, 2020. The patient was taken back to the interventional radiology suite and a guidewire passed from the right percutaneous nephrostomy site down the right ureter and into the presacral space. The guidewire was left in place. Later that day the patient was taken to the cystoscopy suite for the “Rendezvous procedure”. Cystoscopy and rigid ureteroscopy (Uretero-renoscope, Karl Storz Endoscopy-America, Inc., El Segundo, SA) were performed (Fig. 5). The stent was visualized by ureteroscopy and grasped with flexible grasping forceps (Karl Storz Endoscopy-America, Inc., El Segundo, SA). The guidewire was manipulated into the bladder. A nephroureteral stent (Percuflex, El Coyol, Alajuela, Costa Rica) was passed over the guidewire from the skin exit site of the percutaneous nephrostomy into the bladder (Fig. 6).

July 8, 2020. Within 24 h the urine drainages from the closed-suction drain within the presacral space was markedly diminished. The Foley catheter placed at the time of ureteroscopy was maintained in place on constant drainage. The patient was able to resume oral nutrition.

July 14, 2020. The patient was discharged from the hospital with the Foley catheter and nephroureteral stent in place. The closed-suction drain was removed on July 13, 2020. Her sacral incision and bilateral inguinal incisions healed by primary intention.

October 6, 2020. A pyelocystoureterogram was obtained. No leakage was seen. The percutaneous nephrostomy was capped and then removed. The nephroureteral stent was internalized. The Foley catheter was removed. The patient will remain in follow-up to detect ureteral stricture should it form. The ureteral stent is to remain in place indefinitely with stent changes on a 3-monthly basis.

## 3 Discussion

### 3.1 Proper sequence of the steps to complete the rendezvous procedure

In managing a delayed leak from a ureter, it is important that the proper sequence of interventions is followed. The first priority is to assure adequate drainage of urine from the operative site to prevent infection. In the case presented there was a closed-suction drain in place so this happened without an intervention. In most instances, all drains will have been removed because the leak is occurring many days postoperatively or no drain were thought to be necessary. In this instance, percutaneous CT-guided drainage of the urinoma is required. The second step is an attempt at ureteral stenting through the bladder. Usually, this is not successful, but it does in an occasional patient provide definitive stenting of the leak. In this situation a percutaneous nephrostomy is not usually necessary. If ureteral stent placement from below is unsuccessful the next step is a percutaneous nephrostomy. After the nephrostomy is functioning well an attempt to cross the ureteral defect with a stent placed through the nephrostomy should occur. This may be successful but oftentimes it only defines the lowest aspect of the intact ureter. A guidewire is placed through the nephrostomy into the lowest part of the ureter. The fourth step would be a repeat cystoscopy with a ureteroscope available. If the guidewire placed from above can be visualized, it is secured with a grasping forceps and pulled into the bladder. After the ureteral stent spans the defect in the ureter, a tube which functions as a ureteral stent and a percutaneous nephrostomy is inserted.

At this point, weeks or sometimes months of waiting is required. A delay in further interventions should occur until no urine leakage is present. This is a signal that the uroepithelium has covered the stent and fibrosis in and around the ureteral defect has occurred. After drainages of urine have ceased the percutaneous nephrostomy is internalized with a double-J type stent. This stent remains in place for years. Oftentimes it is best that it remain in place for the duration of the patient’s life.

### 3.2 Causes of delayed ureteral leakage

When the urine leak is discovered following the surgical procedure with a closed abdominal incision, it is usually not possible to determine the precise cause of the iatrogenic problem. Chikazawa et al. reported delayed leakage of a ureter after laparoscopic hysterectomy [10]. During this procedure the ureters were maintained at a distance from the operative site by suspension tapes. These authors suggested that the delayed leakage of urine was secondary to ischemia induced by the suspension tapes. Stenting of the ureter resulted in a resolution of the leakage suggesting that a damage to the ureteral sidewall rather than ureteral transection had caused this delayed leak.

A second possible cause for delayed leakage is thermal injury. In our patient who had three prior extensive pelvic surgeries, dense scar tissue was transected using high voltage pure-cut electrosurgical dissection (ball-tip electrosurgical dissection) [11]. Although frequent water cooling of the point of dissection is practiced, considerable heat is generated as a result of the electrosurgical dissection. In the dense scar tissue that was present in this patient’s presacral space heat conduction to the ureter was possible.

Another possibility in our patient was vascular compromise. The ureters had been dissected from an anterior aspect on three prior occasions. Although the ureter was never encountered in the resection of the sacrum along with the mucinous tumor mass, dissection along the posterior aspect of the ureter may have occurred resulting in ischemic damage. This ischemic damage would manifest itself as a delayed leak.

### 3.3 Morbidity associated with reoperative surgery for peritoneal metastases

Cytoreductive surgery combined with a perioperative intraperitoneal chemotherapy is a treatment option for selected patients with peritoneal metastases from colorectal and appendiceal malignancy [12,13]. Also, reoperative surgery for recurrent disease after cytoreductive surgery plus perioperative intraperitoneal chemotherapy has been described [2,3]. Also, third-look procedures, as described in our case report, have been described and associated with success [14]. As the number of interventions increases in these patients, the incidence of fistulas from the gastrointestinal tract and urinary tract increases. Intestinal adhesions and dense scar tissue complicates the safe dissection required to remove the mass of recurrent tumor. The loss of tissue planes is even more severe within the pelvis as compared to the abdomen. This slow and potentially dangerous pelvic dissection led us to resect a presacral mass through access obtained by a partial sacrectomy. This decision resulted in right ureteral trauma and a delayed leak through the distal ureter.

### 3.4 Success expected with rendezvous procedure

Two reports regarding the success of a rendezvous procedure have been reported. Keoghane reported on 18 patients who underwent the rendezvous procedure because of ureteral strictures [15]. Initial success was attained in 16 of his 18 patients. Surprisingly, 7 of these 18 patients could be made stent-free at 3 months. The very high short-term success of the establishment of ureteral function was encouraging. Kawada used the rendezvous technique for postoperative ureteral complications in cancer patients [16]. Initial success was achieved in 17 of his 19 patients. No major complications occurred. Fifty percent of his patients were continued on periodic stent exchanged long-term. Six patients required permanent external drainage or surgical reconstruction. They estimated that 13 out of the 19 patients had a final clinical success (68.4%). These authors emphasize that in patients who had a malignant process requiring rendezvous procedure, long-term success should not be expected. The stenting may indeed be successful but long-term stent-free drainage of the affected kidney may seldom occur because of cancer progression. In this instance large, stiff ureteral stents may provide the best long-term internal drainage free of kidney obstruction.

### 3.5 Alternative approaches to a successful rendezvous procedure

The technology required in our patient from the interventional radiologist and from the urologist was extensive and the cost is high. If a ureteroscopy is not available it is possible to place a wire in the distal ureteral segment. Then this wire can be recovered with a loop placed through the nephrostomy into the proximal ureter. In some patients this retrieval of a wire in the distal ureter using a wire loop technology is possible. Other technologies may be identified in talking with the urologist.

## Declaration of Competing Interest

George Chang, Arshad A. Khan, and Paul H. Sugarbaker have no conflicts of interest to declare.

Saher Sabri is a consultant for Boston Scientific, Medtronic, Philips, and Alucent Medical.

## Funding

Data management and secretarial support provided by Foundation for Applied Research in Gastrointestinal Oncology.

## Ethical approval

Local IRB-approval for this case report was not required: MedStar Health Institutional Review Board has determined that a case report of less than three (3) patients **does not meet the DHHS definition of research** (45 CFR 46.102(d)(pre-2018)/45 CFR 46.102(l)(1/19/2017)) **or the FDA definition of clinical investigation** (21 CFR 46.102(c)) and therefore are not subject to IRB review requirements and **do not require IRB approval**.

This case report is of 1 patient.

## Consent

Written and signed consent was obtained from the patient.

## Author contribution

Paul H. Sugarbaker: study concept or design, data collection, data analysis or interpretation, writing the paper.

George Chang: study concept or design, data analysis or interpretation, writing the paper.

Arshad A. Khan: study concept or design, data analysis or interpretation, writing the paper.

Saher Sabri: study concept or design, data analysis or interpretation, writing the paper.

## Registration of research studies

This study was registered as a case report on the www.researchregistry.com website with UIN 6018. The link to access this is https://www.researchregistry.com/browse-the-registry#home/registrationdetails/5f5fd5e32e72950015927257/.

## Guarantor

Paul H. Sugarbaker, MD.

## Provenance and peer review

Not commissioned, externally peer-reviewed.
